# Non-typeable *Haemophilus influenzae* decreases cilia beating via protein kinase C epsilon

**DOI:** 10.1186/1465-9921-13-49

**Published:** 2012-06-19

**Authors:** Kristina L Bailey, Tricia D LeVan, Daniel A Yanov, Jaqueline A Pavlik, Jane M DeVasure, Joseph H Sisson, Todd A Wyatt

**Affiliations:** 1 VA Nebraska-Western Iowa Health Care System Research Service, Department of Veterans Affairs Medical Center, 4101 Woolworth Avenue, Omaha, NE, 68105, USA; 2 Department of Environmental, Agricultural, and Occupational Health, College of Public Health, University of Nebraska Medical Center, Omaha, NE, 68198-5910, USA; 3 Pulmonary, Critical Care, Sleep & Allergy Division, Department of Internal Medicine, 985300 Nebraska Medical Center, Omaha, NE, 68198-5300, USA; 4 Department of Epidemiology, College of Public Health, University of Nebraska Medical Center, Omaha, NE, 68198-5910, USA

## Abstract

**Background:**

*Haemophilus influenzae* infection of the nasal epithelium has long been associated with observations of decreased nasal ciliary beat frequency (CBF) and injury to the ciliated epithelium. Previously, we have reported that several agents that slow CBF also have the effect of activating protein kinase C epsilon (PKCϵ) activity in bronchial epithelial cells. The subsequent auto-downregulation of PKCϵ or the direct inhibition of PKCϵ leads to the specific detachment of the ciliated cells. METHODS: Primary cultures of ciliated bovine bronchial epithelial cells were exposed to filtered conditioned media supernatants from non-typeable *H. influenzae* (NTHi) cultures. CBF and motile points were measured and PKCϵ activity assayed.

**Results:**

NTHi supernatant exposure significantly and rapidly decreased CBF in a dose-dependent manner within 10 minutes of exposure. After 3 hours of exposure, the number of motile ciliated cells significantly decreased. Direct measurement of PKCϵ activity revealed a dose-dependent activation of PKCϵ in response to NTHi supernatant exposure. Both CBF and PKCϵ activity changes were only observed in fresh NTHi culture supernatant and not observed in exposures to heat-inactivated or frozen supernatants.

**Conclusions:**

Our results suggest that CBF slowing observed in response to NTHi is consistent with the stimulated activation of PKCϵ. Ciliated cell detachment is associated with PKCϵ autodownregulation.

## Introduction

Non-typeable *Haemophilus influenzae* (NTHi) is a non-motile, pleomorphic, gram-negative rod. In individuals with chronic obstructive pulmonary disease (COPD), colonization with NTHi can have significant consequences. When COPD patients are colonized with *H. influenza* in the stable state, they have an increased number of COPD exacerbations. They also have increased symptoms, and increased sputum purulence during exacerbations [1,2].

Part of the detrimental effect NTHi has in COPD may be related to its effects on mucociliary transport. Mucociliary transport functions as an important defense mechanism of the human respiratory tract. Functional mucociliary transport is essential for clearing inhaled pathogens and toxins from the lungs*.* NTHi was shown to contain an undefined factor that causes loss of ciliary activity [3,4]. The mechanism for this slowing is unknown, but has been speculated to involve the cell wall component, lipooligosaccharide (LOS), from *H. influenza.*[5,6] In addition to bacterial products, such as LOS, many agents have been shown to slow cilia, including phorbol myristate acetate (PMA) [7], chronic cigarette smoke exposure [8], pneumolysin [9], acetaldehyde [10], peroxide [11], and neuropeptide Y. [12] However, the precise mechanism of how these agents slow cilia is unknown.

Protein Kinase C (PKC) has been shown to be involved in cilia slowing by many of these agents. However, it is not certain which PKC isoform is responsible for the ciliary slowing. In the case of neuropeptide Y, it was narrowed down to the novel PKC isoforms, which include: PKC δ, ϵ, θ, and η. The authors were unable to narrow the field more however. We have previously shown that modulation of PKC epsilon (ϵ) results in cilia slowing [13]. In this work, we showed that PKCϵ activation led to ciliary slowing and auto-downregulation of PKCϵ led to detachment of ciliated cells. This led us to hypothesize that the ciliary slowing observed with exposure to NTHi may be mediated by upregulation of PKCϵ. Autodownregulation of PKCϵ has also been shown to play a role in the detachment of ciliated cells [13]. We also hypothesized that the autodownregulation of PKCϵ triggered by *H. influenza* may lead to detachment of ciliated cells.

In this series of experiments, we tested these hypotheses using our established model of primary tracheal epithelial cells. These experiments establish that NTHi exposure leads to ciliary slowing and ciliated cell detachment.

## Methods

### Preparation of NTHi supernatant

Nontypeable *Haemophilus influenzae* (NTHi) strain 31P14 was isolated from a COPD patient during an exacerbation of clinical symptoms and was a gift from Dr. S. Sethi (State University of New York, Buffalo) [1]. NTHi cultures were grown overnight in brain-heart infusion broth (Thermo Fisher, Rockford, IL) supplemented with nicotinamide adenine dinucleotide (NAD; 10 μg/mL; MP Biomedicals, Solon, OH) and haemin (10 μg/mL; Alfa Aesar, Wind Hill, MA) in a shaker incubator at 37°C and 225 rpm. Cultures were adjusted to 2 × 10^7^ CFU/100 μl and pelleted by centrifugation at 4°C for 30 minutes at 10,000 *g*. The resulting supernatants were filtered with a 0.2 μm nylon filter and used fresh for experiments. Dilutions of the NTHi supernatants were made using the ciliated cell growth media, M199.

Heat inactivated and frozen supernatants were also tested. Heat inactivation was performed by heating undiluted NTHi supernatants to 95°C for 5 minutes in a thermocycler. The supernatants were allowed to cool and then applied to cell cultures. Frozen samples were prepared by flash freezing the supernatants in liquid nitrogen then allowing them to warm to room temperature.

### Cell culture

Primary bovine bronchial epithelial cells (BBECs) were isolated from bovine lungs obtained from a local abattoir (ConAgra, Omaha, NE) as described previously [14]. Briefly, bronchi were dissected from lungs and digested overnight at 4°C in 0.1% Type IV protease (Sigma, St. Louis, MO) in minimum essential medium (M199 w/Earl’s salts; Gibco, Carlsbad, CA). The lumen of the bronchi was then rinsed with M199 containing 10% fetal bovine serum (FBS; Gibco) to collect the ciliated and basal epithelial cells. The cells were passed through a 70 μm cell strainer (BD Falcon, Franklin Lakes, New Jersey) to collect large clumps of ciliated cells. The clumps of cillated cells were then plated on 60-mm tissue culture dishes coated with 1% type I collagen (Vitrogen; Cohesion, Palo Alto, CA). The clumps of cells were used for ciliary beat frequency analysis. The clumps of cells remain ciliated and beating for over 2 weeks when grown in this manner. However, all of these experiments were performed within one week of harvest. The single cell suspension that passed through the filter was washed in M199 medium, counted with a hemocytometer, and plated (3 × 10^6^ cells) on 60-mm tissue culture dishes coated with 1% type I collagen (Vitrogen; Cohesion, Palo Alto, CA). These cells were used for PKCϵ activity assays. All BBECs were grown in M199 medium containing 10% FBS, 50 U/mL pen-strep (Gibco), and 2 μg/mL amphotericin B (Gibco) in a humidified incubator at 37°C with 5% carbon dioxide.

### Ciliary beat frequency analysis

Live beating cilia were observed in dishes and their motion quantified by measuring ciliary beat frequency (CBF). Changes in CBF were measured using the Sisson-Ammons Video Analysis (SAVA) system as originally described [15]. NTHi supernatant was added to adherent BBECs at concentrations ranging from 0–100%. A baseline CBF was measured at time 0, then the cells were exposed to NTHi supernatant and CBF was monitored every 1 minute for up to 10 minutes, then again at 1, 3, 6, 18, 24 and 48 hours. CBF was expressed in hertz (Hz) for all ciliated cells contained in one field of view. Five unique fields of view per condition (n = 5) were averaged. At least 3 independent experiments were performed.

Motile points analysis is a sensitive way to measure the number of cilia over a large number of cells. To perform motile points analysis, a short video of the cells is recorded. When cilia are moving, the light intensity of each pixel will change rapidly, while the light intensity of non-moving pixels will remain the same. The number of changes in light intensity is recorded, This is the number of motile points. For total motile points analysis, multiple fields of view are analyzed for differential light intensity based on the change of active cilia beating in order to calculate the number of motile points. Motile points were measured at 1, 3, 6, 18, 24, and 48 hrs.

### Dynein dot blot

Detached ciliated cells released into the media supernatant from culture conditions was quantitated using an antibody to ciliary 13S dynein protein as previously characterized [16]. BBEC monolayers were exposed to 50% NTHi supernatants for 1–48 hours. The supernatants were collected and centrifuged to collect any detached cilia and ciliated cells. The pellets were resuspended in 50 mM tris-buffered saline (TBS; pH 7.4) and transferred to nitrocellulose paper using a vacuum apparatus. In addition, 0.1 μg of purified bovine axonemes were loaded as a positive control. The blot was subsequently probed with rabbit anti-13S dynein antibody (1:4000), washed 5 times in TBS with 0.08% tween, and detected with horseradish peroxide-conjugated goat anti-rabbit IgG (1:10,000; Rockland, Gilbertsville, PA). Blots were detected using a SuperSignal West Pico developer kit (Thermo Fisher).

### Protein kinase C epsilon activity assay

BBECs were grown to 70–80% confluency and exposed to 0–100% NTHi supernatant for 1–120 minutes. PKC isoform activity was determined in crude whole-cell fractions of bronchial epithelial cells as previously described [17]. Briefly, samples (20 μl) were added to 40 μl of the following reaction mixture: 900 μM PKCϵ substrate peptide (Calbiochem), 8 μM phosphatidyl-L-serine, 24 μg/ml phorbol myristate acetate (PMA), 30 mM dithiothreitol, 150 μM ATP, 45 mM magnesium acetate, and 10 μCi/ml [γ-^32^P] ATP in a Tris–HCl buffer (pH 7.5). The reactions were incubated at 30°C for 15 minutes. The incubations were stopped by spotting 50 μl of each sample onto P-81 phosphocellulose papers (Whatman, Clinton, NJ). Papers were then washed 5 times for 5 minutes each in phosphoric acid (75 mM), washed once in ethanol, dried and counted in non-aqueous scintillant as previously described [18]. Kinase activity was expressed in relationship to total cellular protein assayed and calculated in pmol/min/mg. All samples were assayed in triplicate and no less than three separate experiments were performed.

### Cell viability assay

Cell viability was assessed by lactate dehydrogenase activity (LDH) assays on the BBEC supernatants, according to the manufacturer’s instructions (Sigma, St. Louis, MO). The supernatant from sonicated BBECs was used as a positive control.

### Statistical analysis

GraphPad Prism statistical package (GraphPad Software, La Jolla, CA) was used to analyze the data. Data was analyzed for statistical significance using one-way ANOVA followed by Tukey post-hoc testing. A *p* value of < 0.05 was considered statistically significant.

## Results

### Supernatants from NTHi slow cilia in a concentration-dependent manner

**Figure 1:**
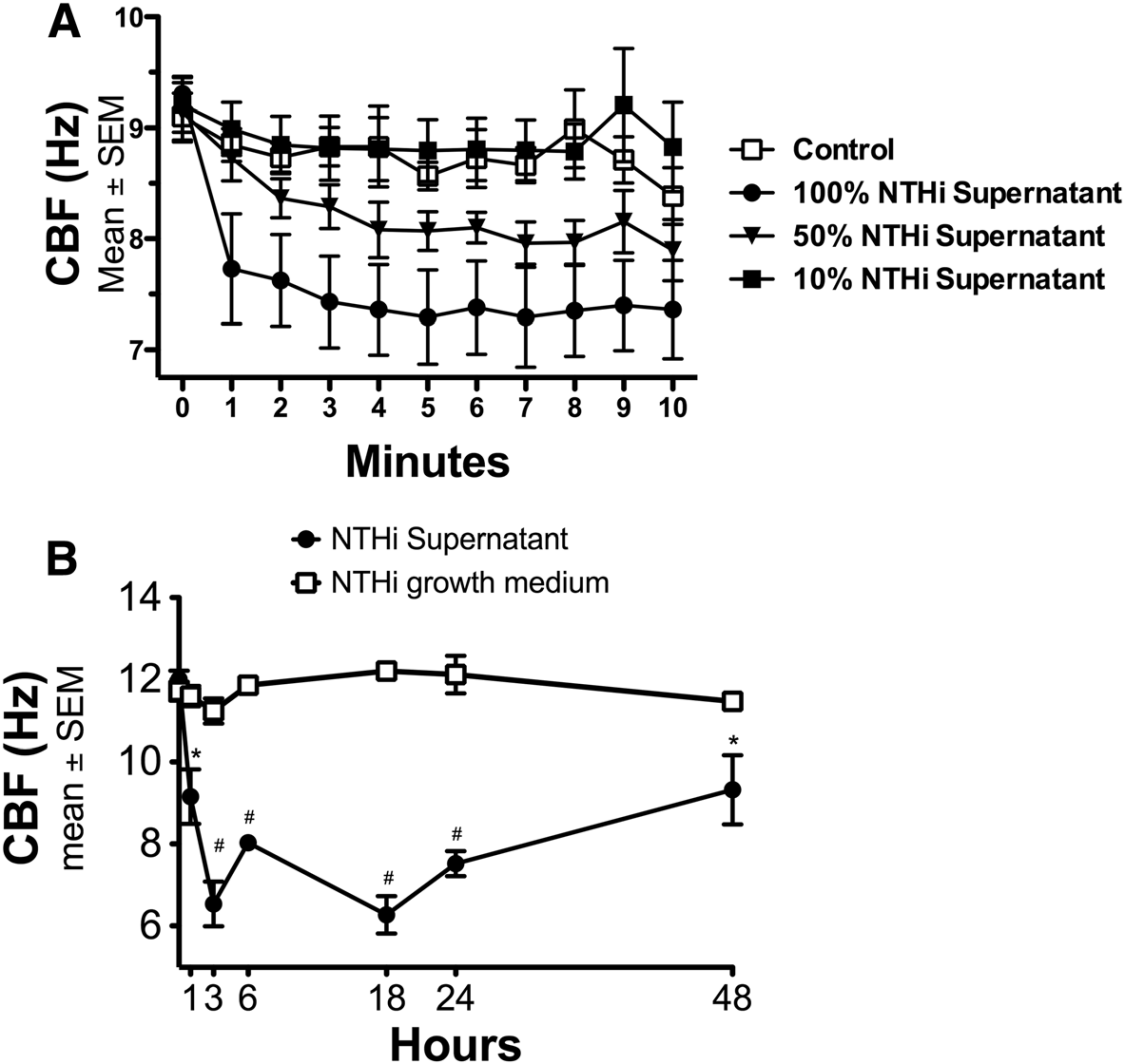
***Supernatants from NTHi slow ciliary beat frequency in a time-and concentration-dependent manner.*A)** Bovine bronchial epithelial cells (BBECs) were exposed to 0%, 10%, 50% and 100% NTHi supernatant for 10 minutes. Ciliary beat frequency (CBF) was measured each minute. NTHi supernatant exposure rapidly slows CBF at the 50% (*p < 0.05) and 100% (#*p* < 0.001) concentrations. There is no difference between 10% and media control. (n = 6) **B)** BBECs were exposed to 50% NTHi supernatant, or 50% NTHi growth medium for up to 48 hours and CBF was measured hourly. The CBF slowing is sustained, with statistically significant CBF slowing at each timepoint compared to control. (*p < 0.05; #*p* < 0.001 vs. media) (n = 8).

To determine the time and concentration dependence of NTHi exposure on CBF, bovine bronchial epithelial cells (BBEC) were exposed to increasing concentrations (0%, 10%, 50%, 100%) of NTHi supernatant at multiple time points. The BBECs exposed to 50% and 100% NTHi supernatant showed a significant slowing of CBF compared to media alone (Figure 1A). Cells exposed to 50% NTHi supernatant demonstrated a 10% decrease (~1 Hz) compared to cells treated with M199 media alone (*p* < 0.05 Figure 1A). This difference was even larger in the 100% NTHi supernatant, with a 20% decrease (~2 Hz) in CBF (Figure 1A). The slowing occurred rapidly, with statistical differences (*p* = 0.05) occurring as early as 1 minute of exposure in the 100% NTHi exposure (Figure 1A). The NTHi supernatant diluted to 10% had no effect on CBF (Figure 1A).

To determine whether the rapid slowing of CBF was sustained over time, BBECs were exposed to 50% NTHi supernatant for 1, 3, 6, 18, 24, and 48 hours. The CBF remained significantly (*p* < 0.05) depressed compared to control at each time point up to 48 hours (Figure 1B). As a control, the cells were also exposed to the NTHi growth media, 50% brain-heart infusion broth/50% M199, which showed no effect on CBF at any time point (Figure 1B). These data indicate that NTHi has a rapid, sustained effect on ciliary beat frequency.

### NTHi supernatant dose-dependently activates protein kinase C epsilon in ciliated cells

**Figure 2:**
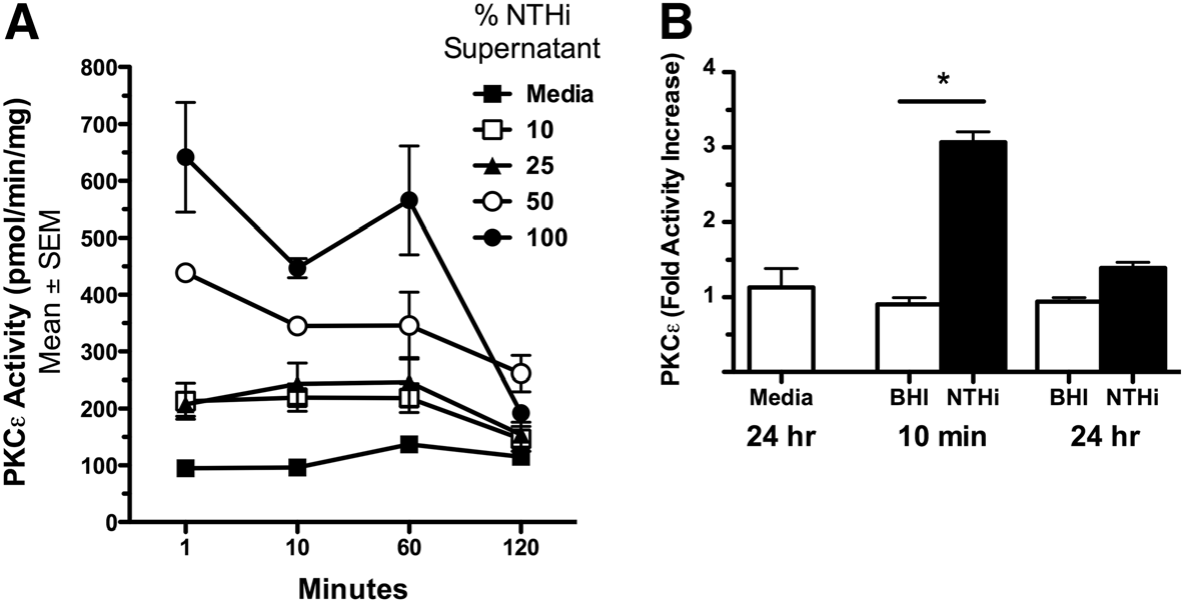
***PKCϵ activity is rapidly stimulated by NTHi.*A)** Bovine bronchial epithelial cells (BBECs) were exposed to 0, 10, 25, 50, and 100% NTHi for 1–120 minutes. The 50% and 100% NTHi rapidly stimulates PKCϵ within 1 minute. PKCϵ activity returns to baseline after 2 hours (*p < 0.05 Media vs. 50% from 1–60 min. p < 0.01 Media vs. 100% from 1–60 min*) (n = 6). **B)** PKCϵ activity remains auto-downregulated at 24 hours (*p < 0.001 vs. Media) (n = 6).

The mechanism of NTHi-mediated ciliary slowing is unknown. Because we have previously shown that activation of PKCϵ plays a role in ciliary slowing [13], we examined PKCϵ activity in response to NTHi supernatants. We observed that PKCϵ activity was rapidly stimulated when BBECs were treated with increasing concentrations (10–100%) of NTHi supernatant (Figure 2A). The maximal stimulation was seen after 1 minute, with 50% supernatant causing a 4-fold increase in PKCϵ activity and 100% supernatant causing a 7-fold increase. An increase in PKCϵ activity remained for up to one hour, then returned to baseline after 2 hours (Figure 2A) and remained auto-downregulated after 24-hour treatment of with 50% NTHi supernatant (Figure 2B). These data demonstrate that NTHi rapidly and transiently activates PKCϵ, a kinase associated with the regulation of cilia slowing. Subsequently, PKCϵ remains auto-downregulated, which has been associated with ciliated cell detachment.

### Heat inactivated or frozen NTHi supernatant fails to activate PKCϵ

**Figure 3:**
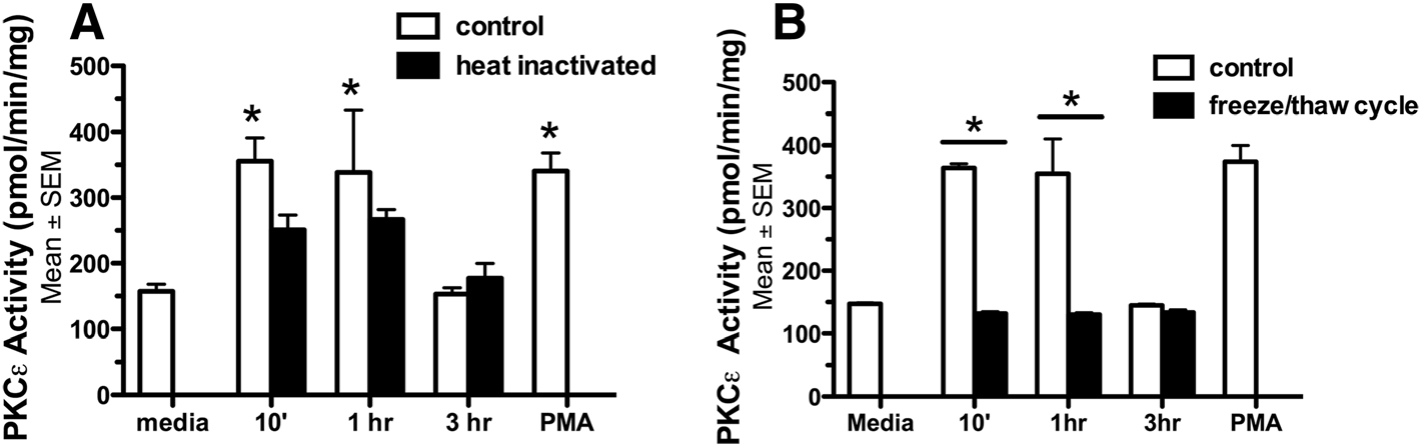
***NTHi supernatant fails to activate*PKCϵ*if it is heat-inactivated or frozen.*A)** Heat inactivation of the NTHi for 5 minutes at 95°C blocks the stimulation of PKCϵ at 10 min and 1 hr (*p < 0.05) (n = 6). PMA is included as a positive control for PKCϵ activation. **B)** Freeze-thawing the NTHi supernatant blocks the stimulation of PKCϵ (*p < 0.05) at 10 min and 1 hr (n = 6).

The component of NTHi supernatant capable of activating PKCϵ is unidentified. To characterize the bioactive component in NTHi supernatants, BBECs were exposed to either freeze-thawed or heat-inactivated NTHi supernatants. BBECs were treated for 10 min, 1 hr, and 3 hr with heat-inactivated (Figure 3A) or freeze-thawed (Figure 3B) NTHi supernatant and PKCϵ activity was assayed. Freeze-thawing or heat-inactivation of NTHi supernatant blocked its ability to activate PKCϵ. Although there is a trend towards increases in PKCϵ in the heat-inactivated samples, the PKCϵ levels are not statistically different than control (*p* > 0.05). Freeze-thawed or heat-inactivated NTHi also failed to slow CBF (data not shown). As a control, NTHi supernatant did not activate PKCα regardless of how the NTHi was pre-treated, i.e. fresh, frozen or heat inactivated (data not shown). These data demonstrate that a temperature and heat labile component contained in the NTHi supernatant is responsible for PKCϵ activation in BBECs and that this response is specific for PKCϵ.

### NTHi supernatant causes a decrease in the total number of motile points

**Figure 4:**
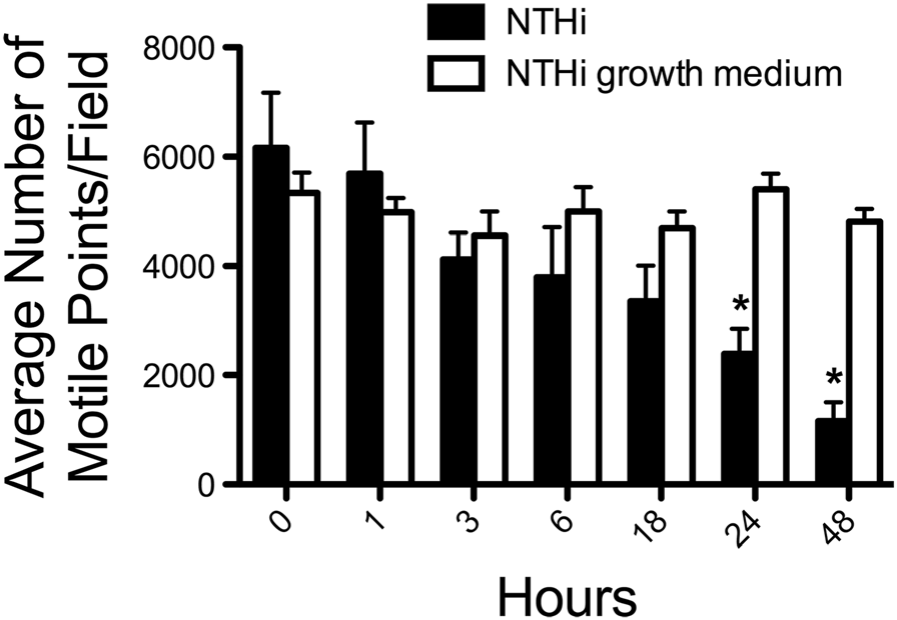
***NTHi supernatant decreases the total number of motile points after 24–48 hours of exposure.*** BBECs were exposed to 50% NTHi supernatant and 50% NTHi growth media alone (Brain-heart infusion broth) for 1–48 hours. The total number of motile points was measured. At 24 and 48 hrs, a significant decrease in motile cilia was detected (*p < 0.01) in the ciliated cells exposed to NTHi supernatents (n = 8).

In addition to CBF, we quantified the number of cilia beating in a microscopic field. Cilliated cells were exposed to 50% NTHi supernatant or 50% NTHi growth medium for 0–48 hours. By 24 hours, a significant decrease in the total number of motile points was detected in the cells exposed to NTHi supernatant (Figure 4; *p* < 0.01). There were no differences in cells exposed to NTHi growth medium alone. The decrease in motile cilia continued up to 48 hours with NTHi supernatant treatment. These data suggest that both the overall average CBF and the mean number of actively beating cilia decrease in response to NTHi supernatant. A decreased number of actively beating cilia can occur under several scenarios, including: 1) the cilia are intact, but immotile 2) the ciliated cells have detached or 3) the cells have undergone lysis or 4) the cilia have been shed, but the cells remain intact.

### NTHi supernatant is not cytotoxic, but results in the detachment of ciliated cells

**Figure 5:**
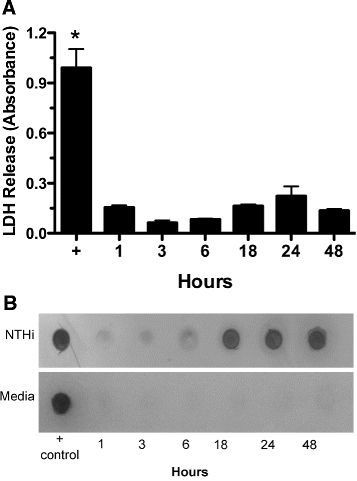
***NTHi supernatant does not cause cell lysis, but it does result in the preferential detachment of ciliated cells.*A)** Media supernatants were collected from bovine bronchial cell cultures treated with 50% NTHi supernatants. Supernatants were assayed for lactate dehydrogenase (LDH) activity. As a positive control, cells were intentionally lysed using sonication. The cells that were exposed to 100% NTHi for 1–48 hours showed no elevation in LDH. This indicates that NTHi does not cause cytotoxicity (*p < 0.05 + control vs. NTHi) (n = 6) **B)** BBECs were exposed to 50% NTHi supernatant for 1–48 hours. The supernatant from these cultures was pelleted to collect any ciliated cells that had detached. Pellets were vacuum transferred and blotted for 13S dynein. Detached ciliated cells were detected at 18–48 hr NTHi supernatant exposure. Positive control is purified bovine tracheal axonemes (0.1 μg) (n = 3).

To determine the cause of the decrease in number of actively beating cilia, we first ruled out cytotoxicity. BBECs were exposed to 50% NTHi supernatant for up to 48 hr, without any significant release of LDH into the media (Figure 5A). The lack of LDH release indicates that cytotoxicity or cell lysis is not occurring. To determine whether ciliated cells were detaching, we measured the ciliary protein 13S dynein in the recovered cell media supernatant. BBECs were exposed to 50% NTHi supernatant for 1–48 hours. We detected increasing levels of the cilia marker protein (13S Dynein) after 18 hours of exposure to NTHi (Figure 5B). In cells exposed to NTHi growth media alone, there was no increase in 14S Dynein staining. These data suggest that NTHi supernatant is not cytotoxic, but does induce the selective detachment of ciliated cells over time, resulting in the decreased number of attached motile ciliated cells in culture.

## Discussion

**Figure 6:**
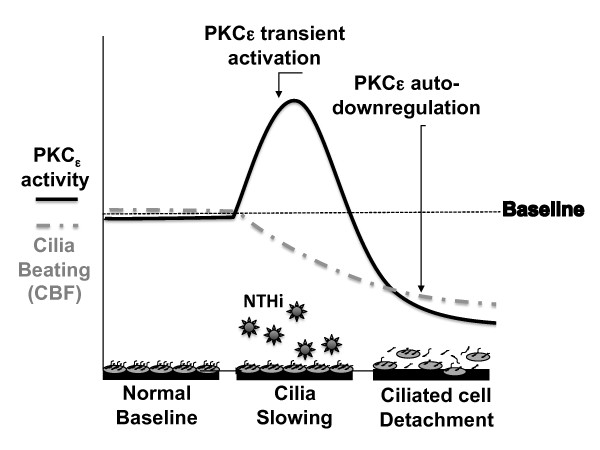
Schematic for the proposed role of PKC epsilon.

We have previously shown that modulation of PKCϵ activity can mediate ciliary slowing and detachment of ciliated cells [13]. Early upregulation of PKCϵ, leads to ciliary slowing. The activation of PKCϵ consequently leads to autodownregulation. It is this autodownregulation that triggers the detachment of ciliated cells (Figure 6). In this series of experiments, we were able to confirm that exposure to NTHi supernatants slows ciliary beat frequency (CBF) in mammalian primary cells. We went on to show that NHTi-mediated CBF slowing and ciliated cell detachment occurs through a PKCϵ-dependent mechanism. In addition, we showed that altering the biological activity of the NTHi supernatant with heat inactivation or freeze-thaw cycles also diminishes the PKCϵ stimulatory effect.

Confirming that NTHi slows CBF was an essential step in our investigations, because technology has advanced significantly since the initial reports were made in 1974 [3]. In that study, ciliary beat was reported on a subjective visal scale of 0–4. Using SAVA, we were able to quantitatively measure CBF. In addition, CBF has never been measured in response to NTHi in our model system of primary bovine bronchial epithelial cells (BBECs). Using this technique, we were able to accurately describe the time and concentration dependence of ciliary slowing in response to NTHi. We observed that CBF rapidly (within 1 minute) decreased in response to exposure to NTHi. This response was sustained, with CBF only returning to baseline after 48 hours. This is an interesting finding because most studies reporting slowing of CBF only make measurements for 2–4 hours.

Perhaps our most interesting finding was identifying the mechanism through which NTHi slows cilia. This is an important finding, because although stimulation of CBF is fairly well understood, very little is known about the mechanisms of CBF slowing. In our experiments, we were able to show that PKCϵ is stimulated very rapidly after exposure to NTHi. This is followed by a rapid decrease in CBF, and auto-downregulation of PKCϵ for up to 24 hours. We have previously shown that pharmacological upregulation of PKCϵ leads to CBF slowing and autodownregulation of PKCϵ leads to detachment of ciliated cells [13]. This same pathway appears to be controlling the NTHi-mediated CBF slowing and detachment of ciliated cells. We were also able to demonstrate that by modulating the bioactivity of the NTHi supernatant through heat inactivation at 95°C or undergoing a freeze-thaw cycle also diminishes the supernatant’s ability to activate PKCϵ.

We also made the novel observation that prolonged exposure to NTHi supernatant leads to detachment of ciliated cells. After 24–48 hours we start to notice a significant decrease in the number of motile points. In fact, the number of motile points drops from approximately 6000 to 1000 after 48 hours of exposure. We were able to determine that this phenomenon was not related to lysis of the cells, but rather to non-cytolytic detachment. This is likely due to the auto-downregulation of PKCϵ. This is consistent with our previous data that PKCϵ auto-downregulation induces the detachment of ciliated cells [13,19].

There are many questions that remain unanswered. It is unclear whether our model best represents an active infection with *H. influenza* or chronic colonization. However, given the relatively short time points that we used in our experiments, we think that our model is likely more representative of an acute infection (as associated with COPD exacerbations) caused by *H. influenza*. In support of this idea is the fact that the strain of NTHi used was isolated from a patient with an acute exacerbation of COPD.

The exact agent in NTHi supernatant that activates PKCϵ and slows CBF also remains unknown. We have shown that its biological activity is diminished by heat-inactivation and freeze-thaw cycles. There are a myriad of substances found in NTHi that could be responsible for its biological effects. Many proteins are degraded by heat inactivation and freeze-thaw cycles. Previous literature suggests that endotoxin may be the agent that slows CBF [3]. However, more recent work has shown that highly purified LPS from *H. Influenza* has no effect on ciliary beat frequency [20]. In addition, Lipooligosaccharide (LOS) [5,6] could also be having an effect. Protein D, an antigen expressed on NTHi, could also play a role in cilia slowing in our model [21,22]. Unfortunately, the supernatant produced by NTHi is a complex mixture containing many proteins, and more sophisticated fractionation of the mixture will have to be performed to determine the exact agent. It is also a possibility that several agents work synergistically to produce the effect.

Taken together, these findings give us a better understanding of how NTHi injures the airway epithelium and the mechanisms that contribute to that injury.

The combination of diminished CBF and detachment of ciliated cells breaks down the protective barrier of the airway epithelium. This likely leads to further damage of the airway epithelium, and could potentially contribute to the clinical decline seen in COPD patients with *H. influenza* colonization or infection.

## Abbreviations

NTHi, *Haemophilus influenzae*; PKCϵ, Protein kinase C epsilon; COPD, Chronic obstructive pulmonary disease; LOS, Lipooligosaccharide; PMA, Phorbol myristate acetate; BBECs, Bovine bronchial epithelial cells; CBF, Ciliary beat frequency; SAVA, Sisson-Ammons Video Analysis system; TBS, Tris-buffered saline; LDH, Lactate dehydrogenase activity.

## Competing interests

The authors declare that they have no competing interests.

## Authors’ contributions

KLB participated in statistical analyses of the data and drafted the manuscript, TDL prepared the NTHi supernatant and participated in statistical analyses of the data, DY carried out cell culture and the PKC epsilon activity assay, and helped draft the manuscript, JAP performed the ciliary beat frequency analysis and participated in statistical analyses of the data, JMD carried out cell culture, the dynein dot blot and the cell viability assay, JHS outlined the ciliary beat frequency analysis and participated in drafting the manuscript, TAW conceived the studies, designed the experiments, participated in statistical analyses and participated in drafting the manuscript. All authors read and approved the final manuscript.
